# The PathOlogist: an automated tool for pathway-centric analysis

**DOI:** 10.1186/1471-2105-12-133

**Published:** 2011-05-04

**Authors:** Sharon I Greenblum, Sol Efroni, Carl F Schaefer, Ken H Buetow

**Affiliations:** 1 Department of Genome Sciences, University of Washington, Seattle WA, USA; 2 Bar-Ilan University, Ramat Gan, Israel; 3 Laboratory of Population Genetics, National Cancer Institute, National Institutes of Health, Rockville MD, USA

## Abstract

**Background:**

The PathOlogist is a new tool designed to transform large sets of gene expression data into quantitative descriptors of pathway-level behavior. The tool aims to provide a robust alternative to the search for single-gene-to-phenotype associations by accounting for the complexity of molecular interactions.

**Results:**

Molecular abundance data is used to calculate two metrics - 'activity' and 'consistency' - for each pathway in a set of more than 500 canonical molecular pathways (source: Pathway Interaction Database, http://pid.nci.nih.gov). The tool then allows a detailed exploration of these metrics through integrated visualization of pathway components and structure, hierarchical clustering of pathways and samples, and statistical analyses designed to detect associations between pathway behavior and clinical features.

**Conclusions:**

The PathOlogist provides a straightforward means to identify the functional processes, rather than individual molecules, that are altered in disease. The statistical power and biologic significance of this approach are made easily accessible to laboratory researchers and informatics analysts alike. Here we show as an example, how the PathOlogist can be used to establish pathway signatures that robustly differentiate breast cancer cell lines based on response to treatment.

## Background

Recent biomedical research has made great strides in unveiling the complexity of human disease. Technological breakthroughs and innovative methodologies now allow a much more detailed account of molecular behavior. Frequently however, such studies yield a plethora of data, with results too complex for traditional analyses designed to identify single genes associated with disease.

Accordingly, many researchers are employing new frameworks to understand disease. One such framework is the concept of pathways - sets of molecular interactions that progress towards a given function. Analysis at the pathway level accounts for some of the data complexity by integrating information from across the entire genome while mirroring real biological processes. Central to pathway analysis is the idea that disruption of the benign behavior of a pathway as a whole, not necessarily a single gene component of the pathway, could be the basis for disease.

The potential benefits of molecular analysis at the pathway level have gained increasing recognition recently, and consequently a number of tools have been developed to visualize pathway structures (Cytoscape [1], Ariadne Pathway Studio [2], PathVisio [3]) and predict novel pathways from experimental data (SRI Pathway Tools [4], GenePath [5]). However, tools to facilitate quantitative informatics-level analyses of established pathways are much less prevalent. To fully explore this promising mode of investigation, a resource is needed that provides a robust and straightforward means to transform large-scale molecular data into meaningful metrics that account for gene relationships at the pathway level.

The PathOlogist is designed to automatically analyze genetic data within the context of molecular pathways. The tool aims to facilitate both a quantitative and qualitative analysis of pathway behavior in a format accessible to both laboratory researchers and informatics analysts.

The PathOlogist uses RNA expression data to calculate 2 descriptive metrics - 'activity' and 'consistency' (see Efroni et al. [6] for motivation and more detailed explanation) - for each pathway in a set of more than 500 canonical pathways (source: Pathway Interaction Database [7]) on a sample-by-sample basis. These two metrics have been shown to be more efficient than individual gene expression at distinguishing samples of different tumor grades and predicting disease outcome in cancer samples [6]. The metrics make use of the structure of gene relationships within in the pathway, rather than treating the genes as simply a uniform set of entities. A pathway is defined as a network of molecular interactions; each interaction consists of one or more input genes, promoters and inhibitors, and one or more output genes. An activity score and a consistency score is calculated for each interaction based on the expression of all input and output genes. Activity scores provide a measure of how likely the interactions are to occur while consistency scores determine whether these interactions follow the logic of the defined network structure. Depending on the nature of the samples, these scores can reveal various types of information. For example, one may compare activity scores calculated from expression data collected at different timepoints to identify functional processes that have been activated or de-activated over time. Comparing consistency scores calculated from sets of tumor and matched normal samples can reveal pathways whose ordinary behavior has been altered by disease.

The PathOlogist facilitates such analyses through a number of features. A clustered heatmap of pathway scores can be generated to provide an overview of the metrics and quickly identify any inherent groupings of samples or sets of pathways that act in concert. The network structure of a pathway and metrics for individual interactions can be viewed as a color-coded graphic, which proves useful for direct comparison of samples and identification of specific areas within the pathway that deviate from normal behavior. Finally, the tool provides an interface for conducting a number of statistical tests to detect associations between pathway scores and additional sample information (for example, disease grade or response to treatment).

## Implementation

The PathOlogist is a MATLAB-based application, which can be run as a GUI in the MATLAB environment or as a standalone executable (with slightly more limited functionality). The objective of the PathOlogist is to transform standard gene or molecule-based data into meaningful, quantifiable information at the pathway level. Our method accomplishes this efficiently using a short sequence of analytic steps designed to maximize fidelity to the original data as well as comparability across studies. The PathOlogist then provides for in-depth analysis of the calculated metrics, through data visualization and statistical tests of association.

### Input

The PathOlogist is designed to analyze normalized abundance data from any gene-based microarray platform, however special features are included to accept Affymetrix data in its raw state as well. The user may upload a set of .cel files reporting probe-level hybridization readings in an arrangement specific to the microarray chip used in the experiment. These .cel files as well as a chip-specific mapping file (easily obtainable from the microarray's commercial website), are the sole input to the PathOlogist necessary to carry out the process of pathway analysis. Once loaded, raw data can be summarized into probesets and normalized using the robust-multichip averaging (RMA) method developed by Irizarry et al [8]. This method is widely used and has been validated as an effective approach in a number of studies [9]. (Note that the RMA-normalization feature is not available in the standalone version.)

Data can also be summarized before input to the tool using RMAexpress http://rmaexpress.bmbolstad.com/). If raw files are not available or if other platforms were used, normalized probeset-based abundance data can be loaded in the form of a textfile.

### Up-Down Normalization

Once the data is defined at the probeset level, a unique algorithm is applied which calculates the probability that each sample is in an 'up' (highly expressive) or 'down' (minimally expressive) state, by fitting the set of intensity readings for a probeset to a mixture of two gamma distributions [6]. The value of this technique is two-fold. First, it effectively places all expression values on a unit scale, allowing direct comparison between different probes, samples, and experiments. This is important for down-stream analysis in which the expression of multiple interacting genes is evaluated in combination. Additionally, this extra normalization tends to significantly reduce noise in highly variable intensity readings, while retaining much more information than a simple 'presence/absence' call.

### Pathway Metrics Calculations

The PathOlogist uses normalized expression data to calculate two descriptive metrics for each pathway selected. For this purpose, a pathway is defined as a connected set of interactions, each consisting of one or more input molecules and one or more output molecules.

#### Source of pathway data

Currently, the PathOlogist uses the PID (Pathway Interaction Database) [7] as the source of pathway structure data. This database is a collection of over 500 canonical pathways, including pathways curated by Nature Publishing Group editors and pathways imported from BioCarta and Kegg. The network structure for each pathway is contained within the tool, and can be updated as new pathways are added to the database.

#### Mapping probes to genes

Mapping probe-level intensity values to molecules within a pathway is accomplished using a platform-specific text file listing the Entrez gene ID associated with each probe. This data is contained within the tool for a number of commonly used platforms. An option also exists allowing the addition of new user-created mapping files, extending the tool's capabilities to virtually any platform.

#### Calculations

**Figure 1:**
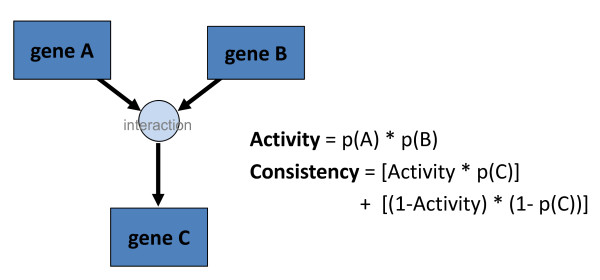
**Activity and consistency scores are calculated for each interaction within a pathway**.

Users can select any subset of samples and pathways to include in metrics calculations. 'Activity' and 'consistency' metrics are calculated for each pathway selected, based on the normalized expression of input and output elements for each sample. Calculations are first performed at the interaction level and then averaged over all the interactions in a pathway to generate a final pathway score. For each interaction, the metrics are calculated as defined in Figure 1, where *p(A) *indicates the probability of gene A being in the 'up' (highly expressive) state. Note that for inhibitory molecules, *1-p(A) *is used instead.

Essentially, activity is a measurement of an interaction's potential to occur, as determined by the expression of input molecules. An activity score of '1' for an interaction indicates that all positively regulating elements are being highly expressed, while inhibitory elements are unexpressed. Consistency scores compare this potential with the actual presence of output molecules, providing an account of deviations from expected pathway logic. Since these two metrics are averaged over all the interactions within a pathway to generate scores for the pathway as a whole, alterations anywhere within the pathway will have the same overall effect.

At the end of the process, each sample will have two scores describing the behavior of each pathway. These scores can then replace individual gene expression values in any desired informatics or statistical analysis.

### Metrics Visualizations

After pathway metrics have been calculated, the PathOlogist facilitates a detailed investigation of the results.

#### Heatmap

A heatmap feature displays activity and/or consistency scores as a bi-dimensionally clustered heatmap. This can be used as a summary view, to quickly identify subgroups within the data. Specific subsets of pathways and samples can be selected for a more directed view as well.

#### Network Graphic

For a specific pathway of interest, a pathway-drawing feature generates a directed network graphic for each of the samples selected, detailing the structure and behavior of the pathway. In the graphic, metrics for individual interactions are displayed visually using node color and size. If desired, the drawing may overlay gene-specific data such as copy number alterations or methylation status. An option also exists to extend the network to include all interactions from other pathways that involve genes within the pathway of interest. Graphics for multiple samples can be compared to identify specific points of differentiation within the pathway. Clicking on any gene within the pathway will link to more detailed information, courtesy of the CGAP Gene database [10]. The network structure and individual interaction metrics can also be generated in text format. (Graphic format not available in standalone version).

### Identifying Important Pathways

The PathOlogist performs statistical analyses to determine the relationship between pathway behavior and sample features such as class, survival, etc. Sample data is entered through a simple copy-paste procedure or by uploading a two-column text file. Four types of analysis are possible:

#### Binary classification

finds pathways whose scores can be used to differentiate two classes of samples (eg. cancer v. normal). For each pathway, a two-sample ranksum test is performed to evaluate the null hypothesis that pathway scores of class A and class B are samples from normal distributions with equal means and variances. Significant pathways are those for which the null hypothesis is highly unlikely, indicating that the pathway behaves differently in these two groups of samples.

#### Linear correlation

finds pathways whose scores correlate well with a continuous variable (eg. response to treatment, measured as concentration of drug required to initiate cell death). For each pathway, the Pearson's correlation coefficient is calculated for the linear relationship between the set of pathway scores and the set of sample data. A p-value is then calculated for each pathway, using a Student's t distribution to evaluate the null hypothesis that the correlation coefficient is zero. Significant pathways are those which show either highly positive or highly negative correlation with the associated variable.

#### Survival

finds pathways that influence sample survival. The set of scores for each pathway are partitioned into two groups using kmeans clustering to minimize the squared Euclidean distance between group centroids. (A minimum group size can be set by the user.) Cumulative survival distributions are calculated separately for these two groups of samples using the Kaplan-Meier algorithm, and a logrank test is performed to evaluate the null hypothesis that the two sample groups are drawn from the same population. Significant pathways are those for which pathway behavior can be used as a marker dividing samples into groups with highly differentiated survival curves.

#### Gene hits targeting

finds pathways whose molecules are the target of some alteration (eg. copy number, mutations). Gene-specific alterations are uploaded as a matrix of logical values describing which of the assayed genes were altered in each sample. For each pathway, a hypergeometric cumulative distribution function is computed for each sample to estimate the probability that genes within the pathway are altered more often than would be expected, given the overall distribution of gene alterations for that sample. An overall p-value for the pathway is calculated by applying a Fisher's Omnibus test to the set of probabilities across all samples. Pathways with a significant p-value are those which comprise a set of genes that are disproportionately altered in multiple samples (although the specific genes altered are not necessarily the same in different samples).

Each test can be performed on all samples or specific classes of samples, and returns a list of all pathways ordered by significance, along with corresponding p-values. These results can be plotted for visual confirmation of association, and then written to text files.

## Results

The power of pathway-level molecular analysis, and the value of the PathOlogist in facilitating such analysis, has been explored using a number of datasets with various features; the results of two such analyses are reported here, using 1) expression and copy number data from a set of 28 cancer cell lines treated with an anti-cancer drug, and 2) expression and survival data obtained from 377 glioblastoma multiforme (GBM) tumor samples, and 10 unmatched normal samples from the publicly accessible TCGA database.

### Pathway Behavior and Drug Sensitivity in Cancer Cell Lines

A recently-analyzed data set assessed the expression profile and drug sensitivity of 28 cell lines maintained by the NCI60 Human Tumor Cell Line Screen [11] as part of NCI's Developmental Therapeutics program. These cell lines were derived from tumors with a variety of cancers and genotypic subtypes. After measuring basal gene expression, each of the cell lines was treated with an anti-cancer drug, and the GI50 concentration (the concentration of drug that causes 50% growth inhibition) was recorded for each. The goal was to identify molecular signatures in the cell lines that would help predict a patient's response to treatment. Additionally, copy number data and other clinical information was available for each cell line.

**Figure 2:**
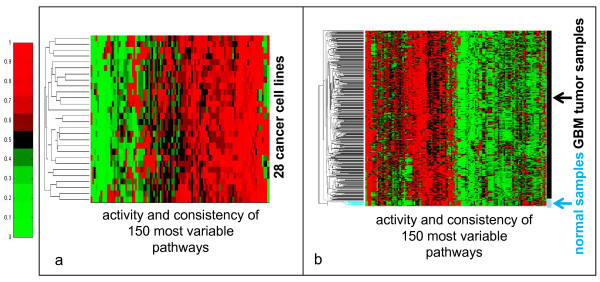
**Bi-dimensionally clustered heatmap of pathway metrics, with separate rows for activity and consistency scores, for a) 28 cancer cell lines, and b) 368 GBM and 10 normal samples**. In b), all 10 normal samples cluster together, as indicated by the colored bars to the right.

The set of .cel files for the 28 lines were loaded into the PathOlogist for RMA normalization. Up-Down Probability normalization was then applied to the RMA data, and the results were used to calculate activity and consistency scores for each pathway in the database. A bi-dimensionally-clustered heatmap of the scores is shown in Figure 2a.

From the heatmap, it appears that the samples do not cluster naturally into separate groups when taking all pathway scores into account. Further analysis is needed to identify specific pathways that influence response to treatment. Thus, GI50 scores (entered as -log[GI50], where GI50 is the drug concentration in moles/liter) for each of the cell lines were loaded into the PathOlogist, and a Pearson linear correlation coefficient was calculated for the set of activity or consistency scores associated with each pathway. An ordered list of pathways and Bonferroni-corrected p-values is shown in Table 1.

**Table 1:** Ordered list of pathways with significant linear correlation between pathway metrics (either activity or consistency) and GI50.

Pathways	Rho	p value*
consistency: il2-mediated signaling events(NCI/Nature)	0.8049	2.18E-04

consistency: trk receptor signaling mediated by pi3k and plc-gamma(NCI/Nature)	0.791	4.91E-04

consistency: cadmium induces dna synthesis and proliferation in macrophages(Biocarta)	0.7511	3.71E-03

activity: toll-like receptor signaling pathway(Kegg)	0.7392	6.32E-03

activity: role of egf receptor transactivation by gpcrs in cardiac hypertrophy(Biocarta)	0.7042	2.60E-02

activity: agrin in postsynaptic differentiation(Biocarta)	0.694	3.80E-02

activity: downstream signaling in naïve cd8+ t cells(NCI/Nature)	0.6869	4.88E-02

*after Bonferroni correction

**Figure 3:**
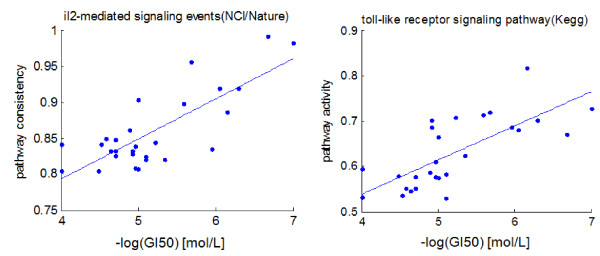
**Scatterplots depicting relationship between GI50 and pathway metrics for two highly correlated pathways**.

From this list we can see that a small set of pathways (n = 7) are significantly correlated with drug sensitivity at the 0.05 level. By contrast, when we shuffled the sample labels and re-performed the correlation analysis 10 times, none of these shuffled distributions produced a single significant correlation after Bonferroni adjustment. Additionally, the PathOlogist was used to depict these relationships in scatterplot form for a few of the top pathways in order to visually confirm the correlation (Figure 3).

**Figure 4:**
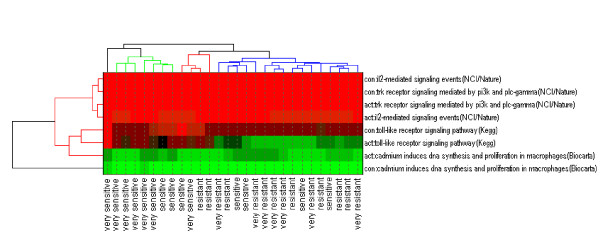
**Heatmap of activity and consistency scores for the four pathways most highly correlated with sensitivity to treatment**. Samples were divided into four equal groups and labeled very sensitive, sensitive, resistant, or very resistant based on their response.

When these pathways are used in a heatmap (as in Figure 4), most of the samples cluster into tight groups based on sensitivity. After correcting for multiple comparison testing (using a Bonferroni adjustment), the most significant pathways are more highly correlated to GI50 than any one probeset.

In addition to identifying pathways whose behavior is differentially altered, it may be useful to gain more insight into what drives these alterations. In order to explore this further, copy number data for each of the cell lines was loaded into the tool, and the PathOlogist was used to find pathways whose components were altered by copy number changes.

**Figure 5:**
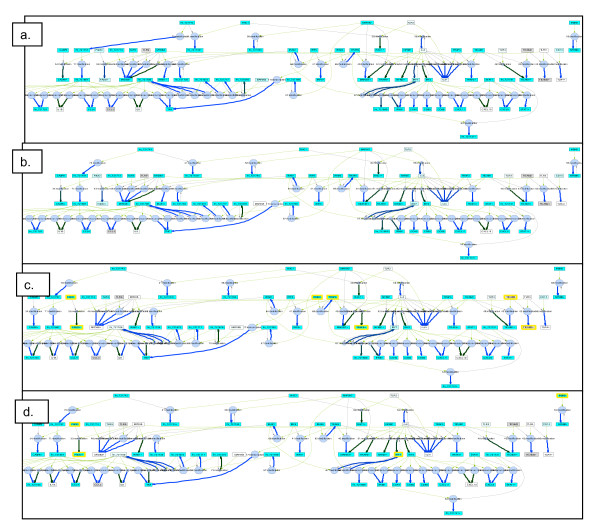
**Network structure of the Toll-like Receptor Signaling' pathway, for two sensitive (a, b) and two resistant (c, d) cell lines**. Molecules with copy number alterations are outlined in yellow.

**Figure 6:**
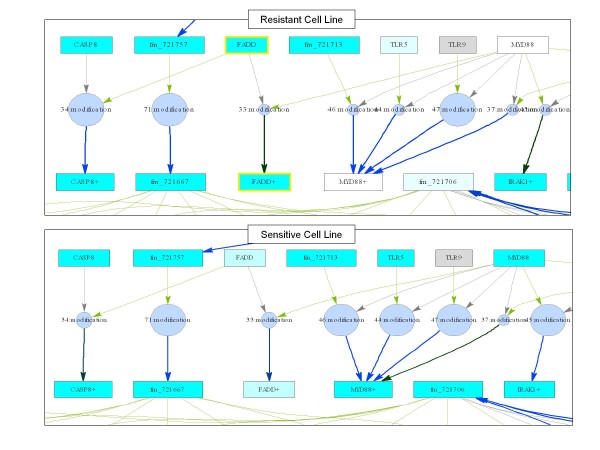
**Portion of the 'Toll-like Receptor Signaling' pathway for two representative cell lines**. Rectangles represent molecules, circles represent interactions. Molecular expression is represented by shading of molecule nodes: white = lowest expression, bright turquoise = highest expression. Molecules with copy number alterations are outlined in yellow. Interaction activity is represented by circle size: large circle = high activity, small circle = low activity. Interaction consistency is represented by arrow color: bright blue: high consistency, dark green: low consistency.

We find that one of these pathways, the 'Toll-like receptor signaling pathway' was also one of the pathways most predictive of drug response. We therefore assessed whether copy number alterations in this pathway are associated with changes in pathway behavior, and consequently, variations in response to treatment. The PathOlogist was used to 'draw' the network structure for each of the samples, depicting molecular expression and copy number, as well as interaction activity and consistency visually in context. Whole networks and zoomed-in views of the network structure for a few representative samples are shown in Figures 5 and 6.

This detailed graphical view is informative in a number of ways. It appears from Figure 5 that the resistant lines are more frequently the target of copy number alterations to molecules in this pathway. Notably though, the same molecules are not altered in each line, although their alteration may lead to the same end result - disruption of pathway function. The pathway-based analysis is able to capture this differentiation in ways that a single-molecule based analysis could not. In addition, a closer look at the network reveals the effect these copy number alterations may have on molecular expression, as well as other specific places in the pathway where expression is differentiated.

### Pathway Behavior and Patient Survival in Glioblastoma Tumors

Publicly available normalized expression data [12] for 377 GBM tumor and 10 normal samples generated using both the Affymetrix U133a platform and the Agilent G4502A platform was downloaded from the TCGA online database.

RMA-normalized data was loaded into the PathOlogist as a text file. Up-Down Probability normalization was then applied to the RMA data, and the results were used to calculate activity and consistency scores for each pathway in the database. A bi-dimensionally-clustered heatmap of the scores for the 150 pathways with the highest sample-wide variance in scores is shown in Figure 2b.

From the heatmap, it appears that the 10 normal samples cluster naturally into a separate group, suggesting that overall pathway-level behavior has been altered in the tumor samples, and that our approach is able to capture these alterations. Further analysis identified specific pathways that are most altered, including many that have been previously implicated in GBM [13] such as ERBB2 signaling (p < 8 × 10^-8^), and TNF-alpha and FAS signaling (p < 7 × 10^-8^). Other pathway-based analyses (Paradigm [14], SPIA [15]) published 15 NCI/Nature pathways with the highest differential behavior between tumor and normal samples. For direct comparison we compiled a list of the 15 top-ranked NCI/Nature pathways from our analysis (Table 2), and find a number of similarities to previously reported pathways, particularly with regard to activity scores. Similarities include pathways involved in Pi3k signaling (p < 9 × 10^-5^) histone deacytelase (HDAC) signaling (p < 9 × 10^-5^), LPA-receptor mediated events (p < 3 × 10^-4^), p38 MAPK signaling (p < 1 × 10^-4^), and hif-1-alpha-mediated regulation (p < 2 × 10^-4^). This overlap suggests that our method is able to find the important features in the data, and identify pathways that are strongly correlated with disease. Furthermore, when we consider the most significantly altered pathways according to consistency scores, many of the pathways we find have extensive experimental evidence [16,17] for involvement in GBM but have not yet been identified by other pathway-centric tools. The RAC1, CDC42, FAS, and PDGFR signaling pathways all appear in the top 15 pathways with altered consistency (yet appear lower in our list according to activity) and have been shown to play a large role in GBM tumor formation. Thus, through the combined use of activity and consistency metrics, our method is able to capture both well-established and previously unreported pathway-level alterations.

**Table 2:** NCI/Nature pathways with highest differential behavior in GBM tumor samples.

Top 15 NCI/Nature Pathways - Activity	p-value
signaling events mediated by hdac class i(nci/nature)	8.56E-05

cellular roles of anthrax toxin(nci/nature)	8.70E-05

hiv-1 nef: negative effector of fas and tnf-alpha(nci/nature)	8.70E-05

nephrin/neph1 signaling in the kidney podocyte(nci/nature)	8.84E-05

class ib pi3k non-lipid kinase events(nci/nature)	8.98E-05

p38 signaling mediated by mapkap kinases(nci/nature)	9.42E-05

hypoxic and oxygen homeostasis regulation of hif-1-alpha(nci/nature)	1.09E-04

hif-1-alpha transcription factor network(nci/nature)	1.16E-04

amb2 integrin signaling(nci/nature)	1.18E-04

signaling events mediated by prl(nci/nature)	1.36E-04

vegfr1 specific signals(nci/nature)	1.36E-04

caspase cascade in apoptosis(nci/nature)	1.39E-04

s1p3 pathway(nci/nature)	1.83E-04

bard1 signaling events(nci/nature)	2.02E-04

lpa receptor mediated events(nci/nature)	2.22E-04

**Top 15 NCI/Nature Pathways - Consistency **	**p-value **

nephrin/neph1 signaling in the kidney podocyte(nci/nature)	8.56E-05

hiv-1 nef: negative effector of fas and tnf-alpha(nci/nature)	8.56E-05

rac1 signaling pathway(nci/nature)	8.56E-05

class ib pi3k non-lipid kinase events(nci/nature)	8.98E-05

cdc42 signaling events(nci/nature)	9.73E-05

bard1 signaling events(nci/nature)	1.05E-04

hypoxic and oxygen homeostasis regulation of hif-1-alpha(nci/nature)	1.12E-04

signaling events mediated by focal adhesion kinase(nci/nature)	1.14E-04

regulation of p38-alpha and p38-beta(nci/nature)	1.40E-04

alpha-synuclein signaling(nci/nature)	1.83E-04

signaling events mediated by hdac class i(nci/nature)	1.97E-04

pdgfr-beta signaling pathway(nci/nature)	2.04E-04

fas (cd95) signaling pathway(nci/nature)	2.39E-04

igf1 pathway(nci/nature)	2.77E-04

noncanonical wnt signaling pathway(nci/nature)	3.09E-04

Within the set of tumor samples, we were also able to identify pathways associated with survival. For each pathway, samples were clustered into two groups based on activity or consistency scores, and a logrank test was performed comparing the Kaplan-Meier survival curve of the two groups. A number of pathways separate samples into groups with significantly different survival curves. Previous analysis with Paradigm identified pathways separating GBM samples into four subgroups, one of which had a significantly increased survival. This group was primarily characterized by upregulation of E2F. Our pathway-based analysis finds that the 'e2f transcription factor network' pathway is the only NCI/Nature pathway for which both activity and consistency are significantly associated with survival. Additionally, we find pathways involving p53, colorectal cancer, and prostate cancer among the most significant.

## Discussion

We believe that the integrated analysis made possible by this tool will prove useful for pathway-based study of biological information. Many tools currently exist that infer networks from expression data. By contrast, the PathOlogist predicts expression based on network structure, and then assesses whether actual gene expression systematically deviates from this prediction. Activity and consistency scores provide a simple yet informative summary of biologically complex behavior in a manner immediately suitable for further statistical analyses. The metrics generated by the PathOlogist can be used to generate pathway signatures associated with clinical data, or identify specific pathways implicated in disease for further insight into disease pathology.

There are however, some limitations to this type of analysis. The PathOlogist can only analyze established pathways, and assumes the accuracy of the pathways in the Pathway Interaction Database. Although the PID is carefully curated to contain only high-quality, well-documented pathways, there is still a certain amount of ambiguity inherent in each pathway's components, structure, and boundaries. On the other hand, the PathOlogist offers a possible check for pathway validity, as it stands to reason that a pathway returning consistently low consistency scores, even for normal samples, could be assumed to lack biologic fidelity. Additionally, since pathway scores are averages of the metrics associated with each interaction within the pathway, small subsets of interactions that have real association to a clinical feature may be overshadowed by other non-correlated interactions, especially in large pathways.

## Availability and Requirements

Project name: PathOlogist

Project home page: ftp://ftp1.nci.nih.gov/pub/pathologist/

Operating system: Windows

Programming language: MATLAB

Other Requirements: The full version of the tool requires a copy of MATLAB as well as MATLAB's Bioinformatics and Statistics Toolboxes. The tool is most stable when using MATLAB version 2009b and later. The standalone version does not require MATLAB but the RMA normalization and single-pathway graphics features are not currently available. A step-by-step manual for use of the PathOlogist is located at ftp://ftp1.nci.nih.gov/pub/pathologist/The%20PathOlogist%20overview.doc (additional file 1).

License: none

Restrictions: None

## Authors' contributions

SG and SE developed and tested the application. SG drafted the manuscript. SE, CS and KB were involved in formulating the initial concepts, and KB coordinated the project. All authors read and approved the final manuscript.
